# Rationales for the Bernese approaches in acetabular surgery

**DOI:** 10.1007/s00068-012-0229-3

**Published:** 2012-09-30

**Authors:** M. J. B. Keel, T. M. Ecker, K.-A. Siebenrock, J. D. Bastian

**Affiliations:** Department of Orthopedic and Trauma Surgery, University Hospital Bern Inselspital, Freiburgstrasse 3, Bern, Switzerland

**Keywords:** Acetabulum, Osteosynthesis, Hip, Geriatric trauma, Pararectus approach, Surgical hip dislocation

## Abstract

**Purpose:**

To present two new approaches to acetabular surgery that were established in Berne, and which aim at enhanced visualization and anatomical reconstruction of acetabular fractures.

**Method:**

The trochanteric flip osteotomy allows for surgical hip dislocation, and was introduced as a posterior approach for acetabular fracture management involving the posterior column and wall. For acetabular fractures predominantly involving the anterior column and the quadrilateral plate, the Pararectus approach is described.

**Results:**

Full exposure of the hip joint, as provided by the trochanteric flip osteotomy, facilitates anatomical reduction of acetabular or femoral head fractures and safe positioning of the anterior column screw in transverse or T-shaped fractures. Additionally, the approach enables osteochondral transplantation as a salvage procedure for severe chondral femoral head damage and osteoplasty of an associated inadequate offset at the femoral head–neck junction. The Pararectus approach allows anatomical restoration with minimal access morbidity, and combines advantages of the ilioinguinal and modified Stoppa approaches.

**Conclusions:**

Utilization of the trochanteric flip osteotomy eases visualization of the superior aspect of the acetabulum, and enables the evaluation and treatment of chondral lesions of the femoral head or acetabulum and labral tears. Displaced fractures of the anterior column with a medialized quadrilateral plate can be addressed successfully through the Pararectus approach, in which surgical access is associated with minimal morbidity. However, long-term results following the two presented Bernese approaches are needed to confirm that in the treatment of complex acetabular fractures the rate of poor results in almost one-third of all cases (as currently yielded using traditional approaches) might be reduced by the utilization of the presented novel approaches.

## Introduction

The gold standard for the treatment of displaced acetabular fractures is the surgical anatomical restoration of the articular surface and stable internal fixation [1]. Poor outcome in the mid-term and long-term follow-up is observed in about 20 % of all operated simple fractures and in 28 % of all operated complex fractures according to the Letournel classification [2, 3]. Fracture pattern, superomedial dome impaction, femoral head damage, increasing age, delay to surgery, and poor quality of reduction (step-off >2–3 mm) are the main causes of poor outcome [4–7]. The quality of reduction depends on whether an adequate view can be obtained of the addressed posterior or/and anterior column of the acetabulum. The Kocher–Langenbeck approach is the gold standard for posterior access [8]. Bernhard von Langenbeck described an incision between the posterior superior iliac spine and the tip of the greater trochanter in 1874. Kocher [9], a professor of surgery in Berne, and a Nobel prize winner for his research on the physiology, pathology, and surgery of the thyroid in 1909, developed the posterior approach further by extending inferiorly along the outer aspect of the thigh, as published in his book* Chirurgische Operationslehre*. The gold standard for the anterior column is the ilioinguinal approach, as described by Letournel [8, 10] in 1960. The iliofemoral approach (Smith–Petersen) for anterior wall fractures or the extended iliofemoral approach for comminuted T-shaped or both-column fractures have occasionally been described, although the latter has a high access morbidity and a high rate of poor results [8, 11].

Acetabular surgery has become more demanding in the last 20 years because this type of surgery has increasingly been performed in patients older than 60 years (the proportion of older patients undergoing this surgery has risen from 10 to 24 %) [12]. In elderly patients, fracture patterns with displacements of the anterior column and quadrilateral plate, roof impaction, comminuted fractures, or marginal impaction in the posterior wall are more frequently observed [12]. The goals of modern acetabular surgery are to optimize the anatomical reduction and the stability of fixation and, in parallel, to reduce the access morbidity. In relation to the treatment of typical fracture patterns in elderly patients with central dislocation, a limited ilioinguinal approach has been reported to reduce the access morbidity, and the modified Stoppa approach, which allows better access to the pelvic brim and quadrilateral plate, has been introduced [7, 13, 14]. Over the past 15 years, the Bernese hip group has established two new approaches in acetabular surgery that aim to achieve better visualization and anatomical reconstruction by allowing a direct view into the hip joint, and to reduce access morbidity. This review presents arguments for surgical hip dislocation and the Pararectus approach as potential standard approaches for the future, as illustrated by four representative cases [15–19].

## Trochanteric flip osteotomy and surgical hip dislocation

**Fig. 1:**
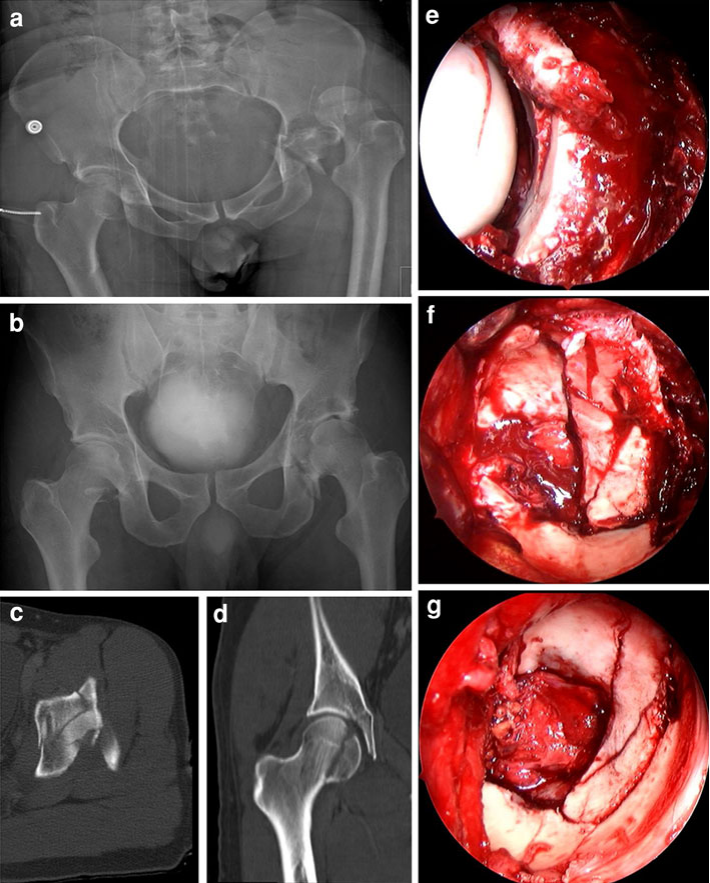

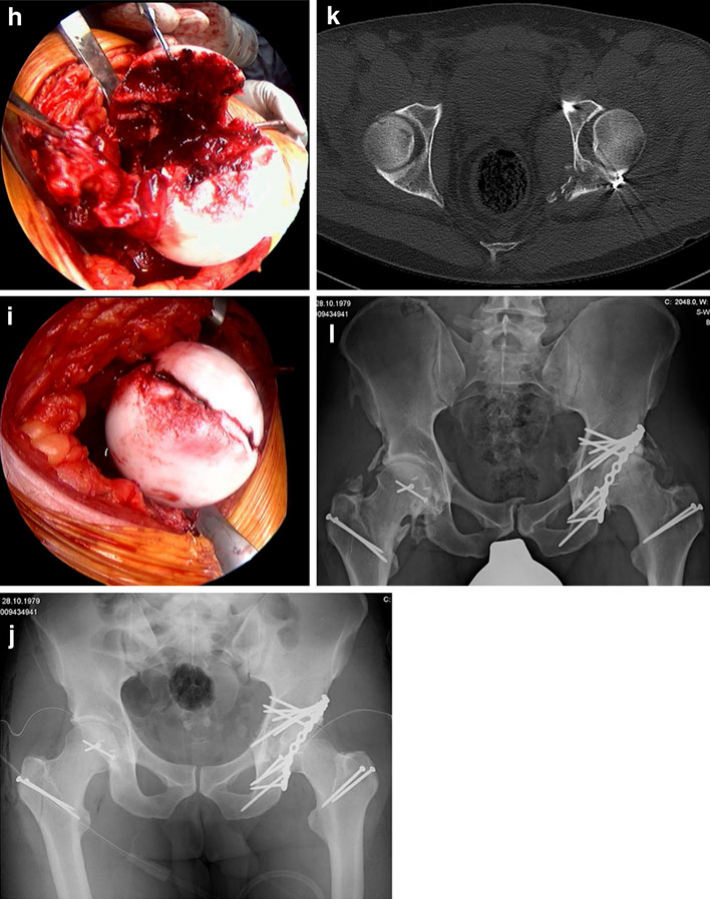
**a** Anteroposterior radiograph of the pelvis of a 30-year-old patient who sustained bilateral posterior hip dislocations due to a work accident before closed reduction. **b** Anteroposterior radiograph of the pelvis after closed reduction. **c** Axial CT scan shows a transverse acetabular fracture with comminuted posterior wall fracture of the left hip. **d** Coronal CT scan demonstrates a femoral head fracture (Pipkin type I) on the right side. **e** Intraoperative view of the left acetabulum through the posterior approach shows marginal impaction and comminuted posterior wall fracture.** f** Trochanteric flip osteotomy and surgical hip dislocation allows complete exposure of the transverse fracture with comminuted posterior wall fracture. **g** Exposure of the acetabulum after anatomic reduction and internal fixation. **h** Intraoperative view of the right femoral head before anatomic reduction and fixation after surgical hip dislocation. **i** Intraoperative view of the right femoral head after anatomic reduction and fixation. **j** Postoperative anteroposterior radiograph of the pelvis demonstrates anatomic reconstructions of the Pipkin I fracture on the *right side* and of the acetabular fracture on the *left side*.** k** Postoperative CT scan demonstrates anatomic reconstructions of the Pipkin I fracture on the *right side* and of the acetabular fracture on the left side.** l** Anteroposterior radiograph of the pelvis 3 years after surgery shows bilateral ectopic ossifications (Brooker grade 2) and moderate signs of posttraumatic osteoarthritis of the left hip of the patient with an excellent clinical result

**Fig. 2:**
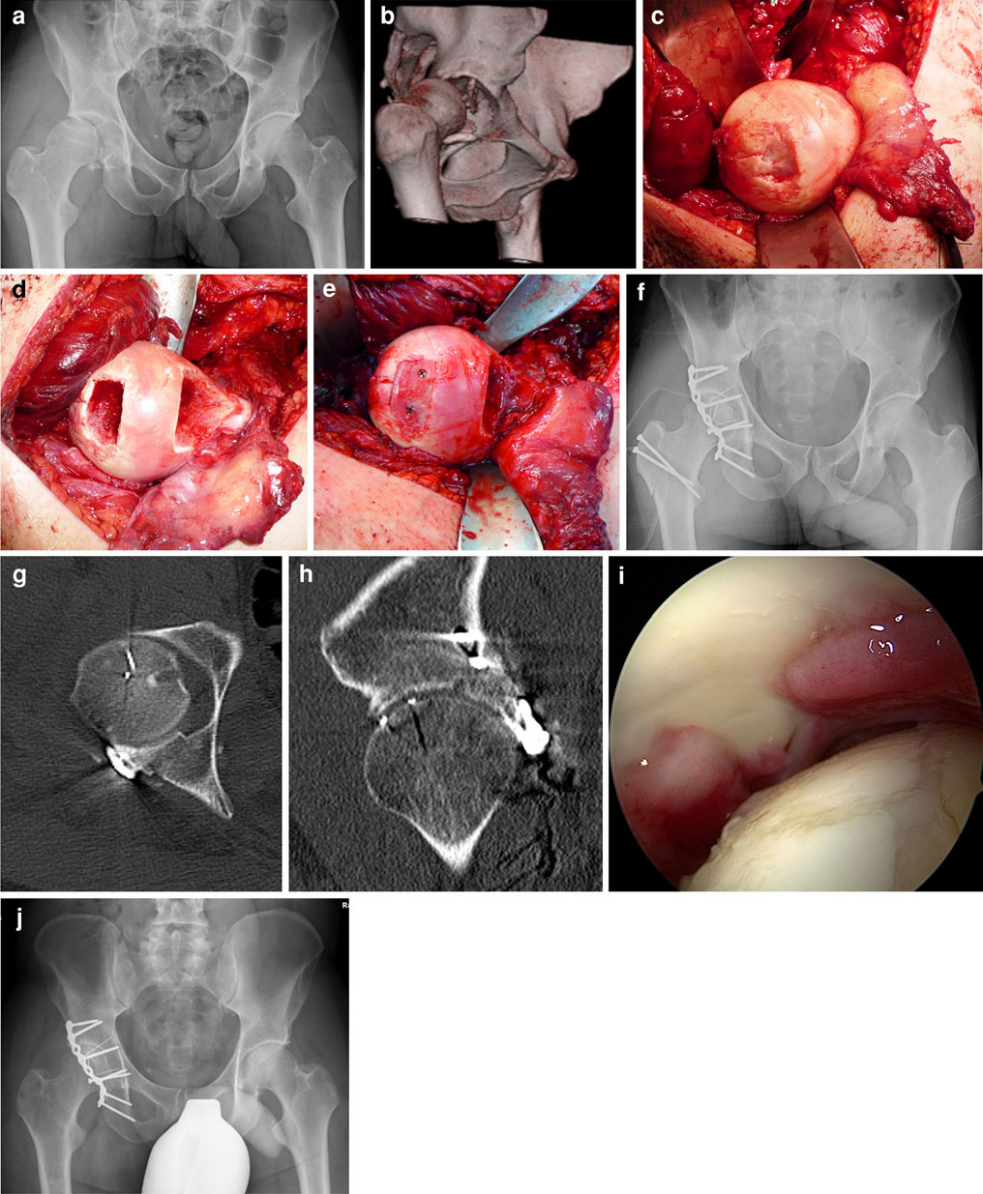
**a** Anteroposterior radiograph of the pelvis of a 44-year-old patient one week after a car accident demonstrates a partially dislocated femoral head on the *right side*, intraarticular bone fragments, and a comminuted posterior wall acetabular fracture. **b** Three-dimensional CT reconstruction demonstrates a partially dislocated femoral head on the *right side*, intraarticular bone fragments, and a comminuted posterior wall acetabular fracture. **c** Intraoperative view of the right femoral head shows the osteochondral defect within the weight-bearing area and the ruptured hip capsule with an intact retinaculum. **d** View of the femoral head after resection of the damaged femoral area and after the harvesting of an osteochondral shell autograft within the femoral head–neck junction. **e** Superior view after reconstruction of the articular surface of the femoral head using the graft and fixation with two 1.5 mm screws. **f** Postoperative anteroposterior radiograph of the pelvis demonstrates anatomic reconstruction of the posterior wall fracture and femoral head damage with osteochondral transplantation. **g** Axial CT scan demonstrates anatomic reconstruction of the posterior wall fracture and femoral head damage with osteochondral transplantation. **h** Sagittal CT reconstruction seven months postoperatively shows complete integration of the graft and the two slightly prominent screws in the femoral head, as well as small cyst formation in the dome as a sign of early osteoarthritis. **i** Arthroscopic view of the acetabular and femoral head cartilages shows moderate chondropathy of the femoral head during hip arthroscopy eight months after trauma from partial removal of the femoral head screws and removal of the trochanter screws. **j** Anteroposterior radiograph of the pelvis 1 year after surgical hip dislocation

The Kocher–Langenbeck approach provides direct visualization of the lateral aspect of the posterior column and the posterior wall. However, access to the posterosuperior and superior wall of the acetabulum is limited. In 1959, Gibson described a more anterior incision that avoided splitting the gluteus maximus muscle and involved dissection in the interval between the gluteus maximus and tensor fasciae latae or the respective gluteus medius muscles [20, 21]. This preserves the neurovascular supply to the anterior portion of the gluteus maximus muscle and extends anterosuperior visualization of the acetabulum. Another modification of the Kocher–Langenbeck approach involves the protection of the short rotator muscles, creating two portals, one between the piriformis and superior gemellus muscles, and the second between the inferior gemellus and the quadratus femoris muscles, while leaving them all attached at both ends [22]. However, although the posterior approach seems to be less invasive with these modifications, manipulations required for visualization, reduction of the posterior column or wall, and superior fixation of the plate lead to stretching and laceration of abductor muscles. Using the trochanteric flip osteotomy makes it easier to visualize the superior aspect of the acetabulum, and the gluteus medius muscle is protected [23, 24]. This approach uses an osteotomy of the greater trochanter, leaving the vastus lateralis and gluteus medius muscles attached to the trochanteric fragment, which is flipped anteriorly. To avoid the risk of femoral head necrosis, the osteotomy runs laterally to the insertion of the short external rotator muscles to protect the deep branch of the medial femoral circumflex artery [25]. The deep branch crosses posterior to the tendon of the obturator externus, protected by the quadratus femoris, and runs cranially, anterior to the other short external rotators. To minimize the risk of a secondary dislocation or nonunion of the trochanteric fragment, and to facilitate the anatomical reduction, a stepped osteotomy of the trochanter was established [26]. Through the Kocher–Langenbeck approach, reduction of the articular surface is performed indirectly using the femoral head as a template, attempting to elevate impacted articular fragments and reducing the cortical bone on the lateral aspect of the posterior column. In the past, the reduction quality was evaluated postoperatively on the three standard plain radiographs (anteroposterior and two Judet 45° oblique pelvic radiographs) [11]. However, Moed demonstrated that residual incongruencies (an offset of >2 mm in 16 % and gaps of ≥2 mm in 78 %) were detected using additional postoperative computed tomography in patients in which the reduction of posterior wall fractures of the acetabulum was rated as “anatomic” according to Matta in 97 % of all cases using plain radiographs [11, 27]. These results indicate that an intraoperative CT scan is needed to control the reduction, or an operative approach allowing direct open reduction of the articular surface is required. Ganz developed the technique of surgical hip dislocation based on knowledge of the blood supply to the femoral head for femoroacetabular impingement (FAI) surgery [15, 25]. After trochanteric osteotomy as described above, the joint capsule is exposed by dissection in the interval between the posteroinferior border of the gluteus minimus and the cranial border of the piriformis muscles. In acetabular fractures with posterior dislocation, the rupture of the joint capsule is integrated into the capsulotomy to preserve the attachment of the wall fragment or, alternately, a Z-shaped capsulotomy is carried out and the femoral head is dislocated posterosuperiorly. The intact capitis femoris ligament has to be cut before dislocation using curved scissors. This full exposure of the hip joint allows different diagnostic and therapeutic options (Figs. 1, 2); [18]. Diagnostically, the damage to the cartilage of the femoral head and the acetabulum can be analyzed. Furthermore, the blood supply to the femoral head can be tested by drilling a 2 mm hole in the femoral head, allowing visual confirmation of pulsatile blood flow. The cartilage lesions of the femoral head are predominantly located anterosuperiorly in the medial sector, and the acetabular chondral lesions are between 11 and 2 o’clock (using 12 sectors, as on a clock face) [18]. Labral lesions were observed with or without hip dislocations and predominantly in transverse fractures. They are located in the posterior aspect of the acetabulum between 8 and 10 o’clock. The labral tears can be refixed by anchors or screws in the case of a small piece of bone [18, 28]. In the case of a multiply ruptured labrum, the damaged part should be resected.

Acetabular fractures associated with femoral head damage or fractures represent a great surgical challenge if poor outcome is to be prevented (Fig. 1). Whereas isolated femoral head fractures can be operated on successfully through an anterior approach (Smith–Petersen approach), combined acetabular fractures of the posterior column/wall and femoral head injuries (Pipkin type IV fractures) are difficult to treat through a single Kocher–Langenbeck approach due to the limited exposure for open reduction and fixation of the femoral head fragments [29]. Implementation of the surgical hip dislocation technique for the treatment of femoral head fractures in our institution 14 years ago increased the rate of excellent or good results to 83 % in our own series [30]. However, significant heterotopic ossifications (Brooker grade 3–4 and/or associated with a significant loss of motion) were noted (8–14 %) depending on whether a single Kocher–Langenbeck approach [11] or a trochanteric flip osteotomy with surgical dislocation of the hip [18] was used. Today, in our treatment concept, the key factors for the prevention of heterotopic ossifications are (1) the use of the trochanteric flip osteotomy in cases of extended posterior wall or superior posterior wall fragments to protect the gluteus medius muscle [24], (2) the debridement of damaged small external rotators and gluteus minimus muscles after traumatic posterior hip dislocation [31], (3) to consequently respect the anatomic dissection of the intervals between the piriformis and the gluteus minimus muscles, as well as the capsule, and (4) the prophylactic administration of indomethacin for two weeks in cases with severe damage to the soft tissues, such as those with traumatic posterior hip dislocation (although radiation therapy has been recognized to be superior to indomethacin in preventing heterotopic ossification after operative treatment of acetabular fractures) [32]. However, radiation therapy might be logistically unfeasible in severely injured patients who are monitored in the intensive care unit, and/or might be undesirable in young patients due to the potential risks of inducing oligospermia, malignancy, or influencing fertility in young women. Thus, radiation therapy is performed mainly in cases requiring revision surgery.

In addition, surgical hip dislocation allows an osteochondral autograft transplantation as a salvage procedure in cases with large femoral head defects after anterior or posterior hip dislocations, as shown in Fig. 2 [33]. The graft is harvested from the non-weight-bearing area of the head–neck junction. Long-term results after this salvage procedure must be analyzed in a prospective collective to assess whether a total hip arthroplasty can be prevented. Furthermore, surgical hip dislocation is helpful for the controlled open reduction of a displaced femoral neck fracture combined with posterior hip dislocation and acetabular fracture (Pipkin type III fracture), in order to preserve the residual attached hip capsule for supplying blood to the femoral head [34, 35]. This technique additionally allows an osteoplasty in the head-neck junction in the case of a nonspherical femoral head causing a FAI in parallel to the restoration of acetabular or femoral head injuries to prevent or delay the development of osteoarthritis [36].

The main advantages of surgical hip dislocation in the treatment of acetabular fractures are the certainty of anatomical restoration of the joint and the safety of optimal extraarticular positioning of the screws (Fig. 1); [18, 37]. Dislocations in the posterior column can be reduced with reduction clamps or the two-screw technique with Jungbluth clamps with or without the femoral head in place. In addition, the technique offers visualization of the entire anterior wall and parts of the anterior column. The reduction of the anterior column in transverse or T-shaped fractures can be facilitated through the use of a bone hook, an elevator, or reduction clamps in the teardrop or from the supra-acetabular region to the anterior horn of the acetabulum. Furthermore, the anterior column lag screw from the posterior aspect of the innominate bone along the column can be perfectly placed in the surgical hip dislocation position [37]. In contrast, using the Kocher–Langenbeck approach, the anterior column is achieved only indirectly through palpation or special instruments, and the extraarticular position of the screw cannot be confirmed.

## Pararectus approach

Over the last two decades there has been a significant increase in acetabular fractures in elderly patients with medial displacement patterns involving the quadrilateral plate and dome impaction [5, 6, 12]. Anatomical reduction and stable fixation in the true pelvis are surgical challenges due to minimal bone stock and limited access through the ilioinguinal approach. A number of different authors have therefore established the modified Stoppa approach for acetabular surgery, which allows better access to the quadrilateral plate [13, 14, 38]. To increase the stability of fixation by the plate on the pelvic brim, a periarticular long screw, the so-called fossa screw, can be placed anterior to the joint in the posterior column [39]. Furthermore, an undercountered medial buttress plate is applied below the iliopectineal line in the true pelvis. The plate is approached through the modified Stoppa approach from the opposite side of the injury. However, a single-incision approach in the vertical midline has only been described in few reports [13, 38]. Frequently a combination of the modified Stoppa approach and the first window of the ilioinguinal approach has been carried out in the range 60–93 % to gain access to the iliac wing [40, 41]. However, the focus of interest, the acetabulum, lies between the first window and the modified Stoppa approach. Therefore, Keel and coworkers [19] developed a new anterior, single-incision intrapelvic anatomical approach. The feasibility of safe dissection and the optimal instrumentation of the pelvis was assessed in cadavers. Clinical evaluation was undertaken in a prospective case series of 20 patients.

**Fig. 3:**
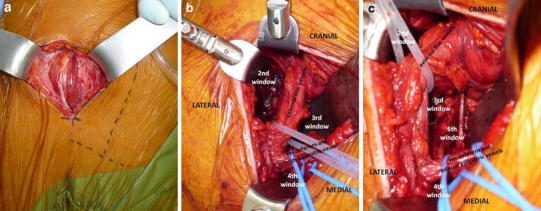
**a** Intraoperative view after a “pararectal” skin incision that starts cranially at the border between the lateral and middle thirds of the line connecting the umbilicus and the anterior superior iliac spine (ASIS), and is directed to the border between the middle and medial thirds of the line connecting the ASIS with the symphysis. After incision of the anterior rectus sheath, the rectus abdominis muscle and the transversalis fascia are visualized. **b** Intraoperative view of the right hemipelvis showing the second, third, and fourth window developed by the Pararectus approach with the external iliac vessels, the vas deferens, and the inferior epigastric vessels indicating the borders of particular windows. **c** Lateral retraction of the external iliac vessels provides access to the fifth window, with visualization of the quadrilateral plate and the obturator nerve

**Fig. 4:**
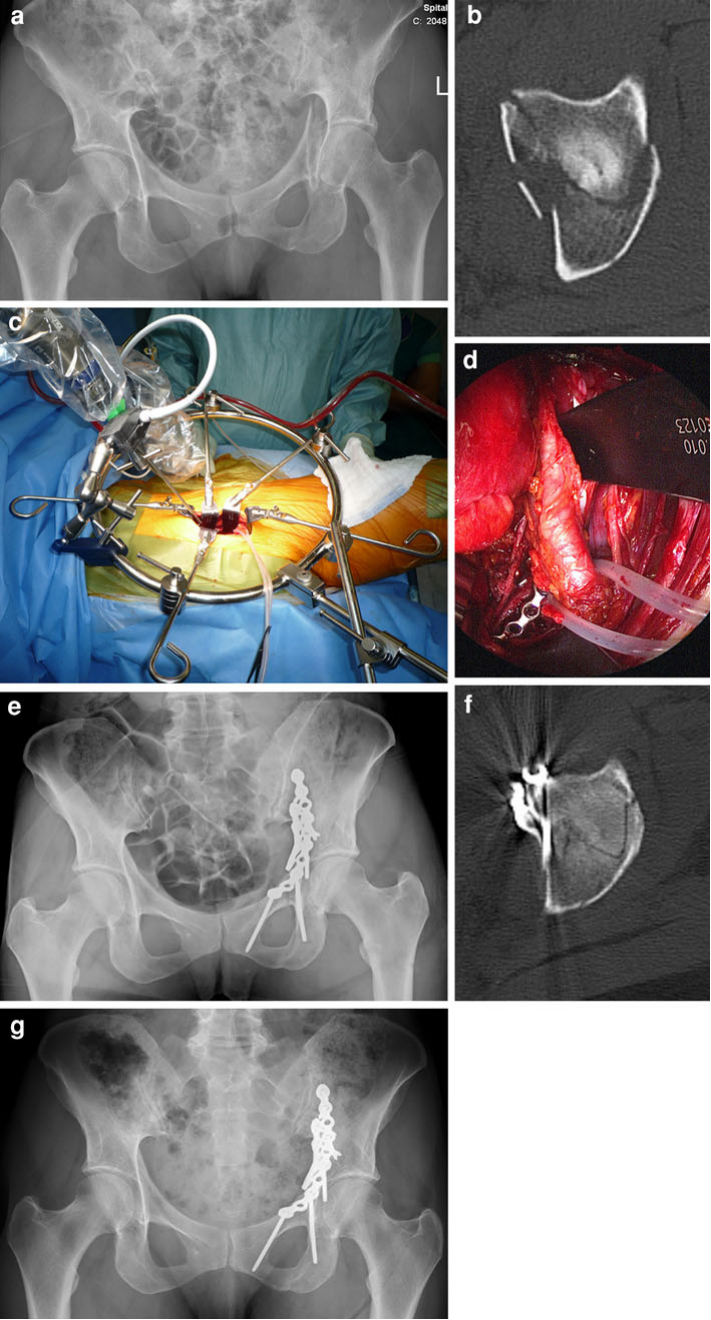
**a** Anteroposterior radiograph of the pelvis of a 56-year-old female patient after a bicycle accident demonstrates a both-column acetabular fracture with a medialized quadrilateral plate. **b** Axial CT scan of the pelvis demonstrates a both-column acetabular fracture with a medialized quadrilateral plate. **c** Intraoperative view of the Pararectus approach and operative setting using the retractor system (Synframe) and endoscope to allow a less invasive approach. **d** Endoscopic view of the obturator nerve (*left*), reconstruction plates on the pelvic brim and quadrilateral plate, and the mobilized external iliac vessels. **e** Postoperative anteroposterior radiograph of the pelvis demonstrates anatomic reconstruction of the both column fracture. **f** Axial CT scan demonstrates anatomic reconstruction of the both-column fracture. **g** Anteroposterior radiograph of the pelvis 2 years after surgery without any signs of osteoarthritis. The patient showed an excellent clinical result after only 6 months postoperatively and is now working as a yoga teacher

**Fig. 5:**
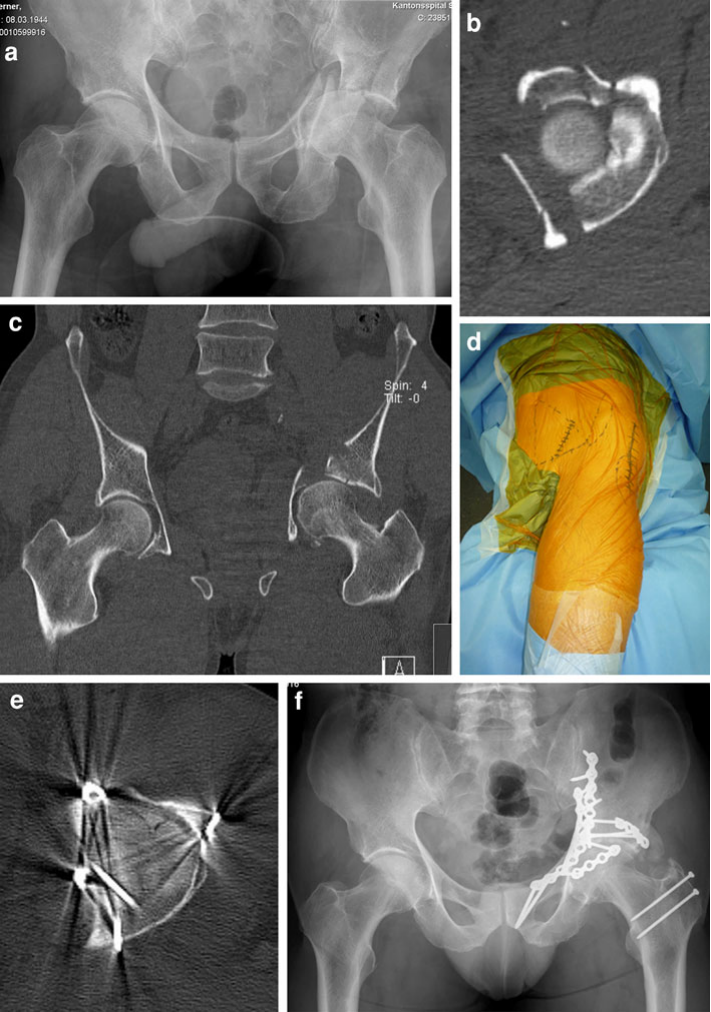
**a** Anteroposterior radiograph of the pelvis of a 67-year-old patient following a bicycle accident that caused a both-column acetabular fracture with central femoral head dislocation, a medialized quadrilateral plate, and dome impression. **b** Axial CT scan of the pelvis demonstrates a both-column acetabular fracture with central femoral head dislocation, a medialized quadrilateral plate, and dome impression. **c** Coronal CT scan of the pelvis demonstrates a both-column acetabular fracture with central femoral head dislocation, a medialized quadrilateral plate, and dome impression. **d** Intraoperative view of the floppy semilateral position that allows simultaneous anterior access by the Pararectus approach and posterior access by the trochanteric flip osteotomy and surgical hip dislocation. **e** Postoperative axial CT scan showing anatomic reconstruction of the left hip. **f** Anteroposterior radiograph of the pelvis 1 year after surgery, with some ectopic ossifications present posteriorly (Brooker grade 2)

We will now describe the novel Pararectus approach according to the published technique [19]. During the learning curve, a general surgeon with experience of vascular surgery should be involved in the dissection of the vessels. Skin incision starts cranially at the border between the lateral and middle thirds of the line connecting the umbilicus with the anterior superior iliac spine (ASIS). The incision is curved and directed to the border between the middle and medial thirds of the line connecting the ASIS with the symphysis. The rectus sheath is incised at the lateral border of the rectus abdominis muscle (Fig. 3). After dissecting the transversalis fascia, the peritoneum is mobilized and retracted craniomedially. Thereafter, five windows can be developed according to the windows of the ilioinguinal approach [8, 10]. In elderly patients with incomplete anterior column fractures, the iliopsoas muscle must be mobilized only rarely, and the first window does not need to be exposed. The external iliac vessels, the inferior epigastric vessels, and the spermatic cord or round ligament are tagged using silastic slings. Aside from the second and the third windows—according to the ilioinguinal approach—the fourth window medial from the epigastric vessels and vas deferens or the round ligament structures are exposed after ligation of the corona mortis (Fig. 3). In addition, after dissection of the internal obturator muscle, medial retraction of the obturator nerve and vessels, and lateral retraction of the vascular bundle, the third and fifth windows are developed with full exposure of the quadrilateral plate (Fig. 3). These windows allow an intraarticular view through the displaced fracture of the quadrilateral plate on the posterosuperior dome impaction fragment using an endoscope. After reduction of the iliac wing fragment, the dome is disimpacted and the periarticular hole is filled with allograft to support the acetabular bone and to prevent a secondary medial displacement of the femoral head. Thereafter, the quadrilateral plate is reduced and fixed by long periarticular lag screws through the plate on the pelvic brim and/or by a buttress plate on the quadrilateral plate (Fig. 4). In the clinical evaluation, the manipulation of the neurovascular structures was safe. Only two lesions to the peritoneum and two regions of minor vascular damage were noted. In the postoperative CT scans, the mean step-off was 0.1 mm and the mean gap 0.8 mm. After a learning curve of about ten cases, and using a special retractor system (e.g., Synframe from Synthes, Oberdorf, Switzerland), the skin incision was reduced from 20 to 8–10 cm. This new approach can be described as “less invasive acetabular surgery” (LIAS), and should be accompanied by less blood loss (Fig. 4). Furthermore, this less invasive approach can be simultaneously combined in a floppy semilateral position with a surgical hip dislocation to achieve anatomical reduction, even in very complex acetabular fractures with severe dome impressions or associated displaced fractures of the posterior wall or column (Fig. 5).

## Conclusions

Surgical hip dislocation allows the full exposure of the femoral head and acetabulum and different therapeutic options to achieve anatomical restoration of femoral head damage or acetabular fractures predominantly involving the posterior wall or column. Displaced fractures of the anterior column with a medialized quadrilateral plate can be addressed successfully through the Pararectus approach, in which case the surgical access leads to minimal morbidity. The two approaches can also be combined to achieve anatomical reduction in comminuted T-shaped or both-column fractures. However, long-term results are necessary to evaluate whether a prognosis of complex acetabular fractures can be optimized through the use of these Bernese acetabular approaches.
